# In Vitro and In Vivo Studies on a Mononuclear Ruthenium Complex Reveals It is a Highly Effective, Fast-Acting, Broad-Spectrum Antimicrobial in Physiologically Relevant Conditions

**DOI:** 10.1021/acsinfecdis.4c00447

**Published:** 2024-08-06

**Authors:** Adam M. Varney, Kirsty L. Smitten, Hannah M. Southam, Simon D. Fairbanks, Craig C. Robertson, Jim A. Thomas, Samantha McLean

**Affiliations:** † School of Science and Technology, Nottingham Trent University, Clifton Lane, Nottingham NG11 8NS, U.K.; ‡ Medical Technologies Innovation Facility (MTIF), Clifton Lane, Nottingham NG11 8NS, U.K.; § Department of Chemistry, University of Sheffield, Brook Hill, Sheffield S3 7HF, U.K.; ∥ School of Bioscience, The University of Sheffield, Western Bank, Sheffield S10 2TN, U.K.

**Keywords:** AMR, ruthenium, combinatorial therapy, ESKAPE, Galleria

## Abstract

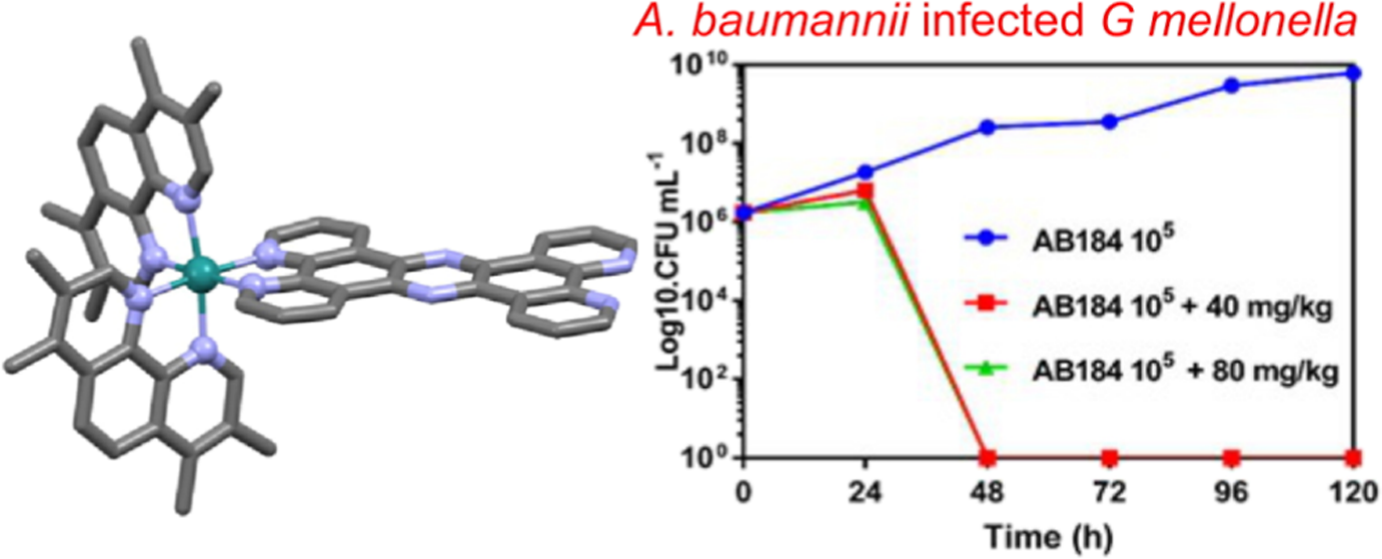

The crystal structure of a previously reported antimicrobial Ru^II^ complex that targets bacterial DNA is presented. Studies utilizing clinical isolates of Gram-negative bacteria that cause catheter-associated urinary tract infection, (CA)UTI, in media that model urine and plasma reveal that good antimicrobial activity is maintained in all conditions tested. Experiments with a series of *Staphylococcus aureus* clinical isolates show that, unlike the majority of previously reported Ru^II^-based antimicrobial leads, the compound retains its potent activity even in MRSA strains. Furthermore, experiments using bacteria in early exponential growth and at different pHs reveal that the compound also retains its activity across a range of conditions that are relevant to those encountered in clinical settings. Combinatorial studies involving cotreatment with conventional antibiotics or a previously reported analogous dinuclear Ru^II^ complex showed no antagonistic effects. In fact, although all combinations show distinct additive antibacterial activity, in one case, this effect approaches synergy. It was found that the *Galleria Mellonella* model organism infected with a multidrug resistant strain of the ESKAPE pathogen *Acinetobacter baumannii* could be successfully treated and totally cleared within 48 h after a single dose of the lead complex with no detectable deleterious effect to the host.

## 1 Introduction

Although the occurrence of antimicrobial resistance was observed almost immediately after penicillin was introduced, the healthy antibiotic developmental pipeline at the time kept medicine ahead of the problem.^1−6^ However, over the last 75 years, the overuse and misuse of antibiotics has created a selection pressure that has accelerated the evolution of antimicrobial resistance, AMR.^7−12^ This process has been acerbated by the COVID-19 pandemic;^13,14^ with up to 47% of inpatients treated for COVID-19 also contracting secondary bacterial infections,^15−18^ the routine use of antibiotics as cotreatments soared.^19^ A multicenter study reported that ∼60% of patients admitted with COVID-19 received antibiotics at admission^20^ while a study involving patients in intensive care due to SARS-CoV-2 infection showed that >90% were cotreated with antibiotics.^21^

Simultaneously, the development pipeline for new antimicrobials is running dry.^22−25^ The newest class of antibiotics in current use was discovered 40 years ago^26^ and the latest WHO annual pipeline report^27,28^ describes only 27 new compounds being clinically investigated as potential treatments for its critical priority pathogens (compared to >5700 compounds currently in development as anticancer therapeutics^29^). More disturbingly, only six of the 27 compounds offer any novelty in terms of mode of action or molecular architecture and merely two of these are being investigated as potential treatments for critical priority Gram-negative pathogens.^28−30^

These alarming circumstances have prompted new investigations into the potential antimicrobial properties of metal complexes.

Strictly speaking such research is a return of attention to this class of compounds; for example, the antimicrobial action of silver was exploited in the classical era and the medicinal use of its salts was well established by medieval times.^31,32^ Indeed, the first ever synthetic antimicrobial, which proved to be a selective and highly successful treatment for syphilis, was the arsenical Salvarsan.^33,34^ And, although it went on to be successfully exploited as an anticancer therapeutic,^35−37^ the biological activity of cisplatin was first reported in the context of its bacteriostatic activity against *Escherichia coli*.^38^ Most relevant to this report was ground-breaking work by the Dwyer group showing that polypyridyl complexes of ruthenium and other transition metals were active against a range of pathogens.^39^ These studies even led to clinical trials on this class of compounds as topical treatments for skin infections.^40,41^

More recently, the Collins and Keene groups resurrected interest in such systems by carrying out a series of investigations into the antimicrobial activity of oligonuclear Ru^II^ and Ir^III^ complexes related to the Dwyer prototypes, discovering that these systems were particularly active against Gram-positive bacteria.^42−44^ This inspired a large number of studies on similar complexes,^45−50^ but again in virtually all such cases, activity against Gram-negative species is considerably lower or entirely lacking.

**Figure 1:**
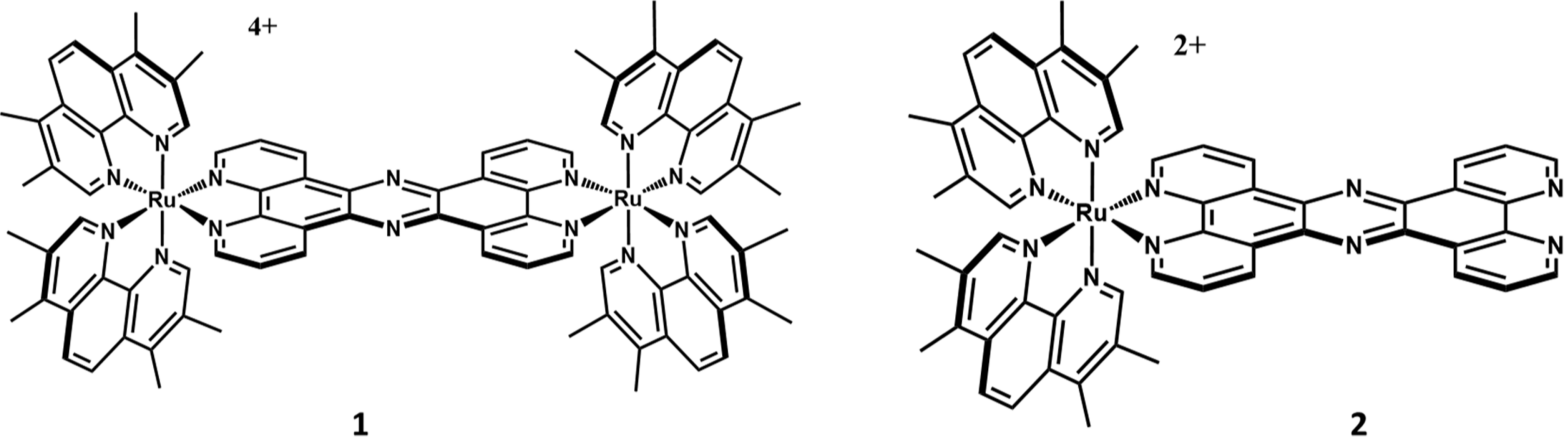
Structures of therapeutic leads discussed in this report. The compounds were studied as chloride salts.

As part of a program of studies on photoactive complexes as sensors and probes,^51−54^ therapeutics,^55−59^ and theragnostics,^60,61^ the Thomas group recently derivatized a dinuclear Ru^II^ complex originally developed as a nontoxic imaging probe for eukaryotic cell nuclei to yield a highly active broad-spectrum antimicrobial, **1**, Figure 1, capable of clearing ESKAPE pathogen infection in vivo.^62,63^ Using the imaging capability of **1**, its mechanism of action was found to involve bacterial cell membrane disruption.^62,64^ In a preliminary follow-on study, we recently showed that **2**, a mononuclear analogue of this lead, is also active but through an entirely different mechanism of action.^65^ Unlike **1**, the mononuclear complex does not disrupt cell–wall structure but is cell permeant and once internalized binds to bacterial DNA. This proposed mechanism of action is distinct from DNA targeting antibiotics such as (fluoro)quinolones that target topoisomerase activity^66^ and the sulphonamides and diaminopyrimidines that target tetrahydrofolate production.^67^ As direct DNA-targeting is a neglected mechanism of action, particularly for a broad-spectrum antimicrobial,^68,69^ herein we report on investigations into the application of **2** as a therapeutic for a range of pathogenic Gram-positive and Gram-negative bacteria in different media and pH conditions, as well as in an in vivo model. Using a selected group of conventional antibiotics and complex **1**, we also assess its potential to be used in a combinatorial therapeutic regime.

## 2 Results and Discussion

### 2.1 Crystal Structure of Complex **2**

**Figure 2:**
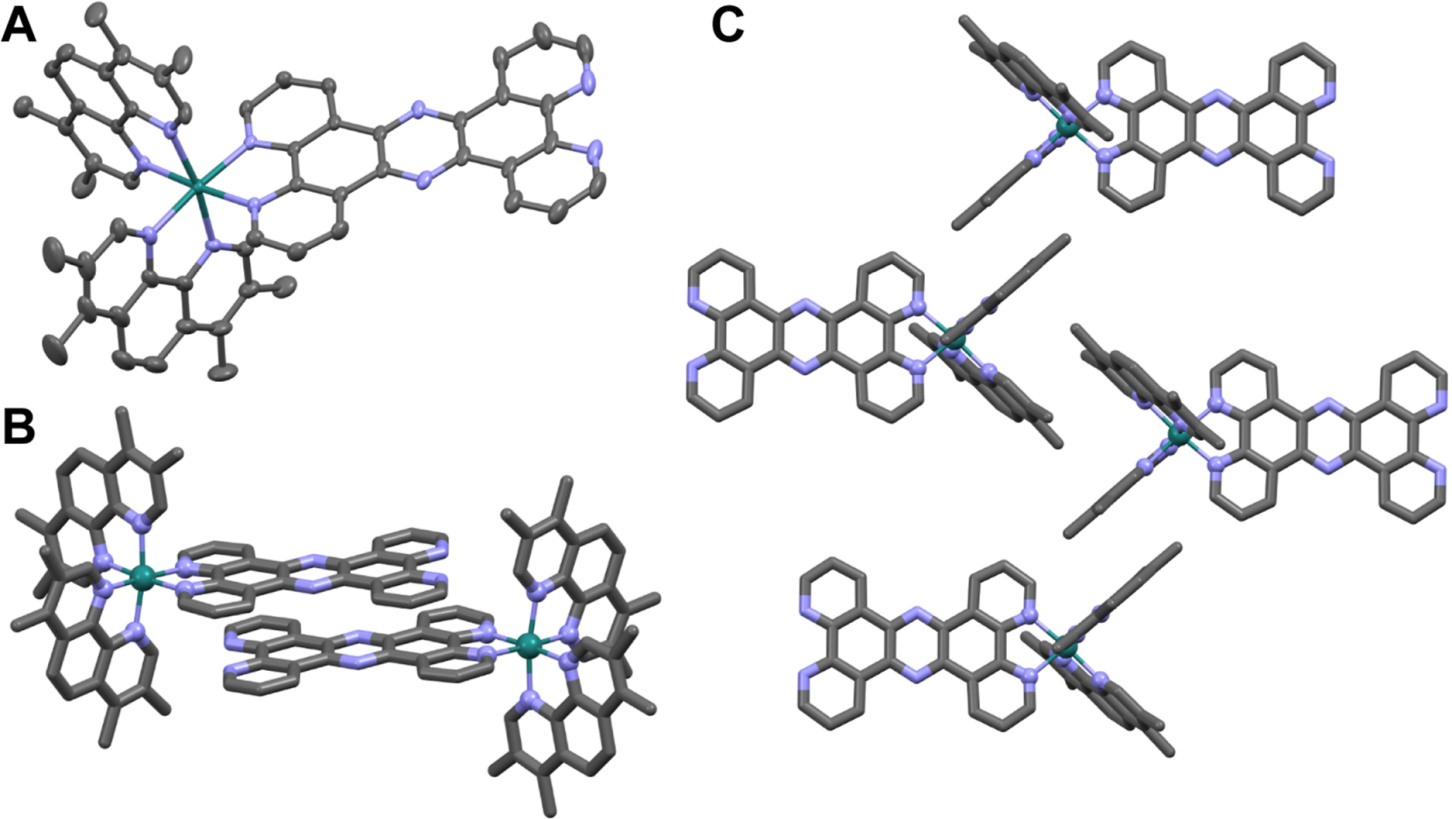
Selected details from the [**2**]Cl_2_ crystal structure. (A) Thermal ellipsoid of the **2** cation. (B) Head-to-tail stacking involving tpphz ligands. (C) Tetramethyl-phenanthroline ligands participate in parallel 4-fold aryl embraces/offset face-to-face interactions. Color key: green = ruthenium, gray = carbon, blue = nitrogen.

X-ray quality crystals were grown by vapor diffusion of diethyl ether into a methanol solution of [**2**]Cl_2_ (Figure 2). The packing of cations within this structure is due to noncovalent motifs involving coordinated aromatic ligands. Pairs of **2** stack through a head-to-tail offset arrangement involving their extended tpphz ligands (Figure 2b), while the tetramethyl-phenanthroline ligands of adjacent cations participate in characteristic parallel 4-fold aryl embraces/offset face-to-face interactions (Figure 2c).

Having confirmed the structure of **2**, its chloride salt was then used for a detailed investigation into its antimicrobial activity.

### 2.2 Antimicrobial Activity in Different Media

Previous studies on metal complex therapeutic leads, including **1**, have shown that apparent activity is often dependent on the medium composition, which may have implications for clinical efficacy. Consequently, we investigated whether this effect was observed for **2**. As it was already established that the complex is active against pathogenic *E. coli* and *Staphylococcus aureus* species,^65^ its efficacy against a panel of previously characterized (Catheter Associated) Urinary Tract Infection ((CA)UTI) clinical isolates in different media was explored.^70^ Three different media were used; glucose-defined minimal medium (gDMM),^62^ and two physiologically relevant media; artificial urine medium (AUM)^71^ and plasma-like medium (PLM).^72,73^ The two clinically relevant media mimic the environment that (CA)UTI strains are likely to encounter during infection and are also standardized to prevent batch-to-batch variation. Selected results from these experiments, which involved a range of (CA)UTI isolates, are summarized in Table 1 (see Table S1 for the full set of bacterial strains tested).

**Table 1:** Minimum Inhibitory and Bactericidal Concentrations of **2** in Multiple Physiologically Relevant Media When Tested Against Clinically Isolated (CA)UTI Bacterial Pathogens^a^

	gDMM		PLM		AUM	
strain	MIC (μM)	MBC (μM)	MIC (μM)	MBC (μM)	MIC (μM)	MBC (μM)
*Escherichia coli* MG1655	1.56	1.56	1.30	25.00	12.50	16.67
*Escherichia coli* EC958	3.13	5.21	1.56	12.50	12.50	33.33
*Klebsiella quasipneumoniae* 18Y000138	5.21	6.25	12.50	50.00	50.00	50.00
*Klebsiella pneumoniae* 18Y001710	1.56	5.21	3.13	20.83	41.67	41.67
*Enterobacter roggenkampii* 18Y001733	3.13	5.21	6.25	37.50	6.25	33.33
*Enterobacter hormaechei* 19Y000094	3.13	8.33	6.25	12.50	25.00	33.33
*Enterobacter hormaechei* 19Y000373	2.08	5.21	5.21	12.50	6.25	25.00
*Escherichia coli* 20Y000092	N/A	N/A	1.56	12.50	3.13	33.33

a Strains were obtained from Nottingham University Hospitals Pathogen Bank, NUH identifies are provided. *N* = 3, standard deviation provided in Table S2.

Comparing the minimal inhibitory concentration (MIC) of **2** in gDMM and PLM showed minimal variation, with MICs remaining low across the different (CA)UTI strains. When tested in AUM, the strains demonstrated less sensitivity to the compound; with *Klebsiella pneumoniae* (18Y000138) having the highest MIC of 50 μM (∼51 mg L^–1^), however, this is still a clinically appropriate concentration of antimicrobial for therapy. That these strains remain sensitive to **2** in multiple clinically relevant media suggests that their efficacy will be unimpaired during the treatment of an infection under physiological conditions. Despite the range of multidrug resistance mechanisms and virulence factors within this panel of clinical isolates,^70^ no strain displayed significantly increased resistance to the complex, suggesting that none of their existing resistance mechanisms cause cross-resistance to **2**. Significantly, this sensitivity was observed across an extended panel of clinical isolates from multiple infection types and genera including *Pseudomonas* sp., *Salmonella* sp., *Citrobacter* sp., *Acinetobacter* sp., and *Serratia* sp., all of which had MICs below 8 μM (8.21 mg/L, Table S1).

### 2.3 Activity Against *S. aureus* Clinical Isolates

**Figure 3:**
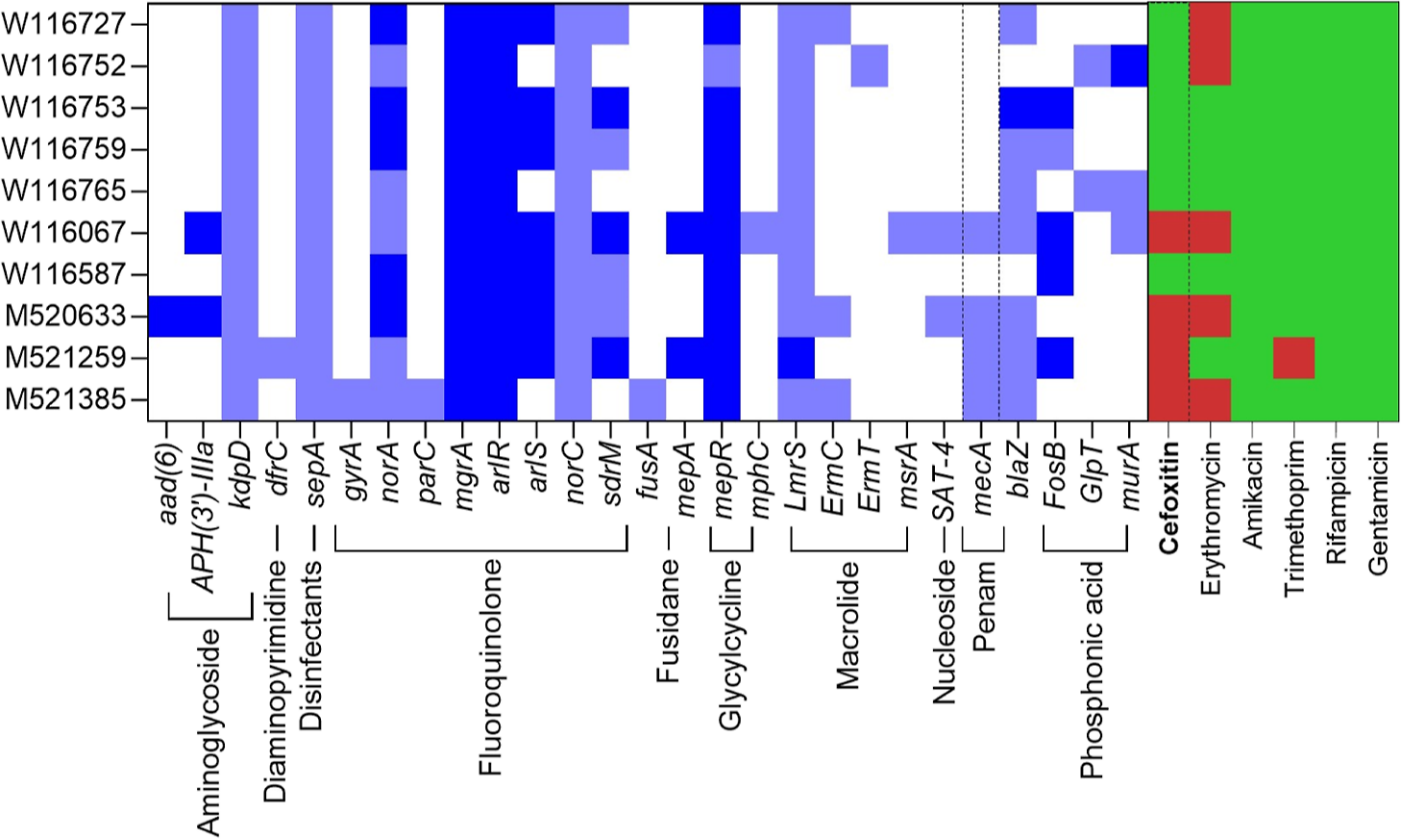
Antibiotic resistance profiles of ten *Staphylococcus aureus* clinical isolates. The whole genome sequences were analyzed for the presence of antimicrobial resistance genes and aligned with phenotypic antibiotic resistance testing data (EUCAST). Dark blue—CARD predicated resistance (perfect), light blue—CARD precited resistance (strict >90%), red—phenotypically resistant, green—phenotypically sensitive. Presence of *mecA* and corresponding cefoxitin resistance are highlighted in dashed boxes.

Resistant strains of *S. aureus* are prominent Gram-positive members of the WHO priority pathogen list. They cause a range of infections, including skin and soft tissue infections, and despite screening and isolation of patients, nosocomial MRSA still poses a serious threat to global healthcare systems. However, apart from some notable exceptions,^68,74−76^ previous studies on ruthenium-based antimicrobials have frequently revealed increased resistances of MRSA strains compared with methicillin sensitive strains.^42,77,78^ Consequently, having confirmed that **2** displays a breadth of activity across Gram-negative pathogens in physiologically relevant conditions, we then investigated its potency across a range of clinical isolates of *S aureus*. To assess the effectiveness of **2** against both methicillin sensitive and methicillin resistant strains of *S aureus*, a panel of ten clinically isolated strains were obtained and assessed phenotypically and genotypically for antimicrobial resistance. Of the ten isolates, four were classified as methicillin resistant and six as methicillin sensitive based on the presence of the *mecA* gene, which was subsequently confirmed by a cefoxitin disk diffusion assay (Figure 3). In fact, the strains display resistance to a spectrum of different antibiotics, and the Comprehensive Antibiotic Resistance Database Resistance Gene Identifier suggests that a range of antimicrobial resistance genes are involved (Figure 3).

As the sensitivity of *S. aureus* to **2** in defined minimal medium has previously been demonstrated,^65^ herein the sensitivity of the ten clinical isolates, including MRSA strains, in both chemically defined medium and a physiologically relevant plasma-like medium was investigated (Table 2).

**Table 2:** Activity of Complex **2** Against a Range of Clinically Isolated AMR Strains of *S. aureus* Including MRSA

		chemically defined medium		plasma-like medium	
strain/ID^a^	origin	MIC (μM)	MBC (μM)	MIC (μM)	MBC (μM)
*S. aureus*	ATCC 29213	1.56	2.34	0.20	2.60
*S. aureus* USA300	LAC JE2^b^	1.56	1.56	0.20	6.25
*S. aureus* W116727	wound	1.56	1.56	0.20	3.13
*S. aureus* W116752	wound	1.43	0.91	0.24	12.50
*S. aureus* W116753	wound	0.91	1.56	0.37	6.25
*S. aureus* W116759	wound	1.56	1.56	0.24	4.43
*S. aureus* W116765	wound	1.56	6.25	0.33	5.21
MRSA W116067	wound	1.56	1.56	0.20	6.51
*S. aureus* W116587	wound	1.56	1.56	0.33	6.77
MRSA M520633	patient swab	1.30	1.56	0.39	3.39
MRSA M521259	patient swab	1.56	1.56	0.20	4.17
MRSA M521385	patient swab	0.78	0.91	0.20	4.17

a Strains were obtained from Chesterfield Royal Hospital, hospital identifiers are provided. *N* = 3 SD is presented in Table S4 represented in Supporting Information. MRSA classification was determined via presence of *mecA* gene.

b See ref (79).

In contrast to **1**, it was found that Gram-positive *S. aureus* shows a greater sensitivity to **2** than the Gram-negative (CA)UTI pathogens tested in the same conditions. All clinically isolated *S. aureus* strains showed similar levels of sensitivity to **2**, despite their differing AMR and virulence profiles (Figures 3 and S1 and Table S3), and crucially the sensitivity of *S. aureus* isolates to **2** was the same in both MRSA and methicillin sensitive strains offering evidence that its efficacy would be unaltered in a clinical setting against a wide variety of pathogens and highlighting its potential in the treatment of MRSA infections.

### 2.4 Complex **2** is Active Against Pathogens in Early Exponential Growth Phase

Although the inhibitory and bactericidal effectiveness of **2** was estimated using standard MIC methodology, this requires low initial turbidity cultures and dilution from a stationary phase; yet, in a clinical setting, antibiotic treatment is often administered when the bacterial load is high and/or the infective isolate is actively growing. To better understand the effectiveness of **2** against actively growing bacterial cultures in a more clinically relevant setting, we exposed clinical isolates in early exponential growth phase to complex **2** at 1× and 10× their minimal inhibitory concentrations.

**Figure 4:**
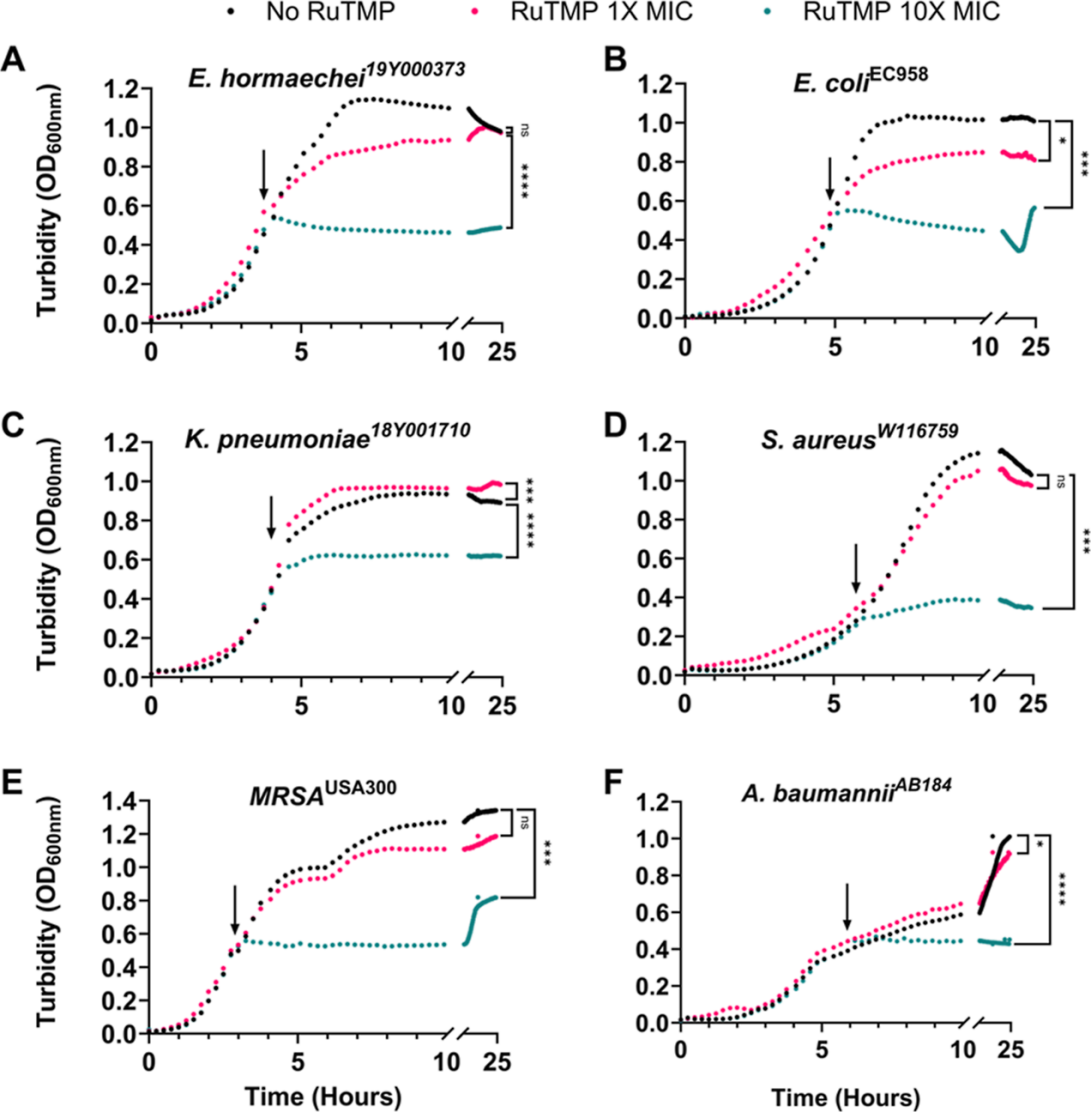
Complex **2** shows growth inhibition of multiple pathogens at early exponential growth phase. Cultures were grown to early exponential phase 37 °C and 475 CPM shaking in 24-well plates in gDMM (A–C), CDM (D,E), gDMM + 1× MEM (F). Upon reaching early exponential phase (OD_600 nm_ 0.4–0.5), 1× or 10× the MIC of the complex or vehicle control was injected and growth monitored at 15 min intervals across the 24 h time course. *N* = ≥ 6. One-way ANOVA was performed on final culture turbidity’s with Dunnett’s multiple comparisons test. Significance levels: not significant (ns, *p* ≥ 0.05), * (*p* < 0.05), ** (*p* < 0.01), *** (*p* < 0.001), and **** (*p* < 0.0001).

In these conditions, unlike previous studies on **1**,^63^ there was no delayed inhibition of growth upon exposure to **2** (Figure 4). Here, exposure to ten times the minimal inhibitory concentration of **2** caused a significant decrease in final carrying capacity of all tested species (*p* < 0.001). This demonstrates the rapid activity of **2** in preventing growth of both Gram-positive and Gram-negative bacterial pathogens.

### 2.5 Bacterial Sensitivity to Complex **2** Across a pH Range

Physiological conditions encountered during infection provide a range of pH environments, including: skin pH (∼4–6), infected wounds (∼7), urine (∼4.5–8), UTI urine (∼8.5–9), and the rapidly changing environment of the gastrointestinal tract: from the highly acidic stomach (∼1.5–3) with variable pH through the duodenum (∼6), small intestine (∼6–7.4), cecum (∼5.7), and rectum (∼6.7). Organisms such as *E. coli* are likely to encounter these conditions during infection, particularly *E. coli* in the urinary and gastrointestinal tracts. Consequently, if complex **2** is to be delivered systemically, it will likely encounter a range of pH’s. Therefore, the inhibitory and bactericidal activity of **2** against a clinically isolated *E. coli* was investigated over a wide pH range. *E. coli* EC958 was chosen for experimentation as it displays a general pH tolerance, and significant genotypic and phenotypic data are available for this strain, making it an appropriate model organism.

**Figure 5:**
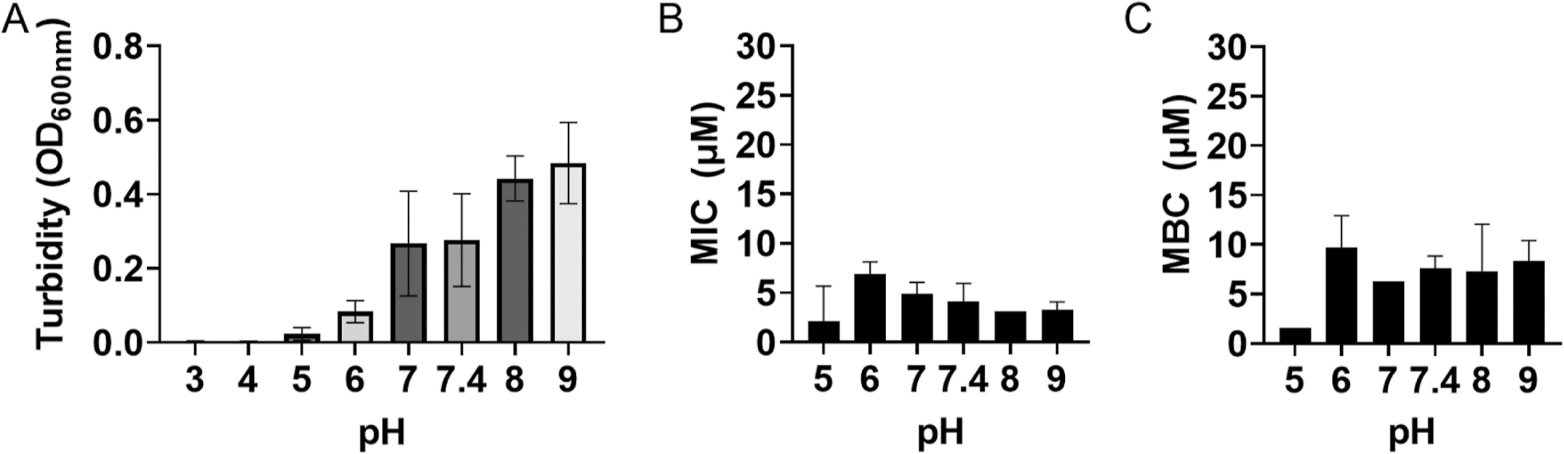
Complex **2** remains inhibitory and bactericidal against *E. coli* EC958 across a broad pH range (5–9). (A) Turbidity readings (OD_600 nm_) were taken from a 96-well plate after static incubation in gDMM at 37 °C for 18 h. (B) Minimal inhibitory concentration testing was performed at under the same conditions as (A) through a pH range of 5–9. (C) Minimal bactericidal concentration assays were performed by spotting 10 μL of all MIC wells with no growth onto Mueller–Hinton agar and incubation at 37 °C for 24 h prior to identification of growth. *N* ≥ 3 ± SD. No statistically significant difference was observed between pH’s 6–9 for either the inhibitory nor bactericidal assays (one-way ANOVA: *P* > 0.05).

The range of *E. coli* EC958 acid tolerance was confirmed by overnight growth. The strain grew between pH’s 5–9, with decreasing turbidity observed in increasingly acidic conditions and no growth detected at pH 4 or below (Figure 5a). We therefore tested inhibitory and bactericidal concentrations between pH’s 5–9. The minimum inhibitory and bactericidal concentrations of **2** against *E. coli* EC958 remained stable at all pHs tested suggesting that the complex will retain activity in diverse physiological conditions (Figure 5b,c).

### 2.6 Combinatorial Effects with Conventional Antibiotics

Dual antibiotic treatments are frequently used to treat severe infections, such as bacteremia and sepsis, particularly when caused by Gram-negative pathogens, including the ESKAPE pathogens. Effective combination therapies should use antimicrobials that are not antagonistic but display additive activity or synergy to assist in the clearance of infections. If antagonistic combinations are used, not only will such treatments likely fail but they could also facilitate the emergence of further antibiotic resistance. We therefore assessed the therapeutic action of **2** in vitro as a component of combination therapies with commonly used antibiotics. Five antibiotics were selected for testing against *E. coli* EC958 using EUCAST standard guidelines to determine the precise clinical sensitivity of the strain in Mueller–Hinton broth and gDMM, which was used as a reference for the synergy assay. *E. coli* EC958 was clinically resistant to β-lactam and quinolone antibiotics and exhibited high levels of resistance to cephalexin, ampicillin, and ciprofloxacin (Table S6). When tested in gDMM some decreased resistance to these three antibiotics was observed. *E. coli* EC958 was categorized as sensitive to Meropenem and nitrofurantoin in accordance with EUCAST guidelines.

Following MIC testing, **2** was assessed for antagonism or synergy in combination with the conventional antibiotics and dinuclear complex **1** by assessing their fractional inhibitory concentration, FIC. Checkerboard assays, performed in gDMM, showed that all tested combinations of antibiotics with **2**, including its dinuclear analogue, were additive (FIC index 0.5–4), with no evidence of antagonism in any of the combinations (Table 3). Notably, the combination of Meropenem and **2** had the lowest FIC index (0.58) exhibiting pronounced additive effects close to synergy (≤0.5). The observation of an additive effect on cotreatment with **1** and **2** confirms that the two complexes display different mechanisms of action. Overall, these encouraging observations indicate that **2** offers great potential for use in combination therapy and would not interfere with the administration of extant antibiotics.

**Table 3:** Assessing the Therapeutic Interaction of Complex **2** with the Antibiotics Used in Combination Therapies

antibiotic	FIC index	antagonistic/additive/synergistic
Meropenem	0.58 ± 0.22	additive
Ampicillin	1.01 ± 0.00	additive
CEF	1.00 ± 0.00	additive
Ciprofloxacin	0.68 ± 0.19	additive
Nitrofurantoin	1.08 ± 0.19	additive
**1**	0.91 ± 0.16	additive

### 2.7 In Vivo Efficacy Studies of Complex **2** Against the ESKAPE Pathogen *Acinetobacter baumannii*

Although the in vitro studies presented above demonstrate that **2** clearly displays potential as a broad-spectrum antimicrobial active in a range of physiologically relevant conditions, these results may not be fully indicative of the activity of the compound in vivo. This is why, prior to their move into human studies, the efficacies of leads are usually studied in mammalian or insect models. Commonly, infection models are studied within rodent or zebrafish models prior to moving to large mammalian models. However, recently there has been an increase in the use of *Galleria mellonella* (greater wax moth larvae) in toxicology screening and as an infection model.^80−83^

Apart from lower costs and greater convenience of *G. mellonella* compared to traditional mammalian models, it presents ethical and logistical advantages in the context of the move toward the reduction, refinement, and replacement, 3Rs, of animal testing. One further attraction of *G. mellonella* is that, unlike other nonvertebrate models, but similar to mammals, it has an innate immune system comprising humoral and cellular responses.^80,84−86^ This means there is a good correlation between bacterial virulence in mammals and the *Galleria* model, which has been successfully exploited to study the pathogenesis and treatment of bacterial infections and give information about potential dosing for future preclinical and clinical studies. In a recent report, we described the use of *G. mellonella* as an infection model to study the antimicrobial efficacy of **1** against multidrug resistant *A. baumannii* infections^87^ and as a previous toxicology screen^65^ had shown that **2** is nontoxic to *Galleria* up to concentrations of at least 80 mg kg^–1^—we set out to investigate the potential of the complex to act as an in vivo treatment for *A. baumannii* and to compare the reported activity of **2** to that of **1** in the same in vivo infection model.

*A. baumannii* is a member of the ESKAPE group of bacterial pathogens causing hospital acquired infections and carbapenem resistant strains were classified by the WHO as “Priority 1: critical” in urgent need of research and development of new antimicrobials.^88,89^ An extensive range of antibiotic resistance genes are present within the *A. baumannii* pan genome, and multidrug resistant strains are widespread across the globe. In addition, extensively drug-resistant and pan drug resistant strain prevalence is also increasing at an alarming rate. Indeed, in many regions of the world, carbapenem resistant *A. baumannii*, CRAB, strains are now the most commonly encountered form of this pathogen.^90^ Clinical manifestations of CRAB range from urinary and respiratory tract infections to bacteremia and meningitis and such infections often lead to high mortality rates (>30%).^91,92^ Furthermore, thanks in part to COVID-associated superinfection, nosocomial CRAB is increasingly becoming associated with ventilator acquired pneumonia.^90^

**Figure 6:**
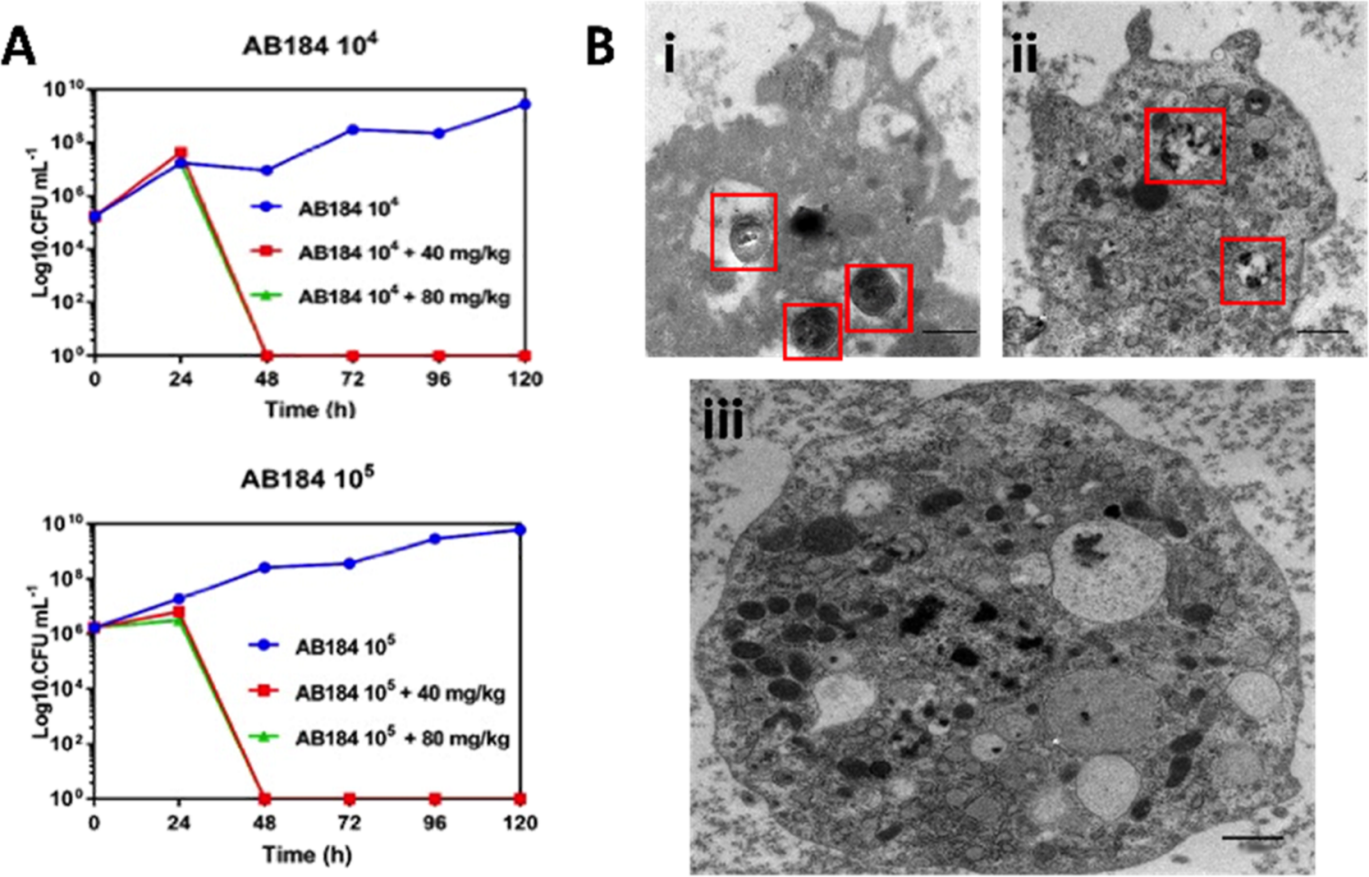
(a) *G. mellonella*-based infection model. Larvae were first exposed to *A. baumannii* at 10^4^ CFU mL^–1^ (top) or 10^5^ CFU mL^–1^ (bottom), then injected with 40 mg kg^–1^ or 80 mg kg^–1^ of **2**. (b) TEM of hemolymph extracted from *G. mellonella* at 24 and 48 h post inoculation with *A. baumannii* and **2**. Protocol: Larvae were injected with bacteria in their right pro-leg. Treated *Galleria* received a dose of the complex 30 min later in their left pro-leg. Larvae were incubated for 120 h at 37.5 °C.

The *G. mellonella* model we have developed uses a multidrug resistant CRAB, the AB184 strain, which is representative of one of the most common *A. baumannii* clonal groups in both the UK and the USA.^87,93^ In the current studies, larvae were injected with AB184 at two different concentrations (10^5^ or 10^6^ CFU mL^–1^). After 30 min, infected larvae were then treated with a single dose of either 40 or 80 mg kg^–1^ of **2** and results were compared to untreated controls. As in previous studies, apart from mortality, the effects of exposure to **2** on the health of larvae was monitored through activity and melanization scoring over the course of the experiment. To ensure that the observed difference between survival of the coinjected larvae and the bacteria injected controls was a direct result of infection clearance, the bacterial load in the larval hemolymph was monitored over the 120 h experiment (Figure 6a).

Survival curves associated with these experiments (Figure S2) were plotted for treated infected larvae and compared to water and AB184 only controls; controls using uninfected larvae that were treated with **2** were also carried out, and as observed in a previously reported toxicity screen, these revealed no detectable toxic effects at either concentration.

In contrast to the untreated control group of larvae, where continual exponential increase in AB184 bacterial colonies was observed over 120 h, either dose regimen of **2** resulted in the total eradication of AB184 infection in all treated larvae between 24 and 48 h (Figure 6a).

Strikingly, this rate of clearance is considerably faster than that observed for **1**, which in identical conditions took 96 h to clear the same *A. baumannii* infection. Furthermore, whereas previous statistical analyses have confirmed that AB184 is pathogenic to *G. mellonella* at concentrations 10^5^ CFU mL^–1^ and above, log-rank *t* tests performed on the survival curves for AB184 infected larvae (40 and 80 mg kg^–1^) treated with **2** showed no significant difference between the treated larvae and the water controls (concentrations 10^5^, 10^6^: 40 mg kg^–1^*P* = 0.0554 and 0.0549 and 80 mg kg^–1^*P* = 0.1385 and 0.1380). This analysis indicates that the complex is an effective treatment for larval CRAB infection in this model organism. Further evidence confirming the clearance of the infection was provided by the intrinsic imaging properties of the complex.

As complex **2** contains an electron dense ruthenium center, it is an effective contrast stain for transmission electron microscopy, TEM, and using this property, hemolymph from infected larvae treated with **2** was extracted at 24 and 48 h post treatment (Figure 6b). The resultant images confirmed that **2** is preferentially taken up by *A. baumannii* cells, as they displayed more pronounced contrast than host cells. Although *A. baumannii* cells were detectable within the extracted hemolymph and in hemolymphocyte cells to 24 h, the hemolymphocyte cells had phagocytosed the bacteria in a manner that is analogous to macrophages and neutrophils. At 48 h, not even dead bacterial cells were observed within hemolymph and hemocyte cells and the hemocyte cells had a healthy morphology. In comparison, although complex **1** also effectively treated infection at similar concentrations total clearance of *A. baumannii* from larvae was only observed after 96 h of exposure.^87^

## 3 Conclusions

Although the increasingly urgent global health challenge of treating AMR pathogens is revitalizing fundamental research into the development of antimicrobials, entirely new molecular scaffolds toward these goals are still severely lacking. This is reflected in the fact that despite the increased activity in this arena, there is still a dearth of genuinely innovative broad-spectrum antimicrobials entering the development pipeline. In this study, we carried out a detailed assessment into a recently identified potential therapeutic lead for AMR pathogens. These studies show that the complex is highly active against a range of multidrug-resistant strains of Gram-negative (CA)UTI and ESKAPE pathogens, including clinical isolates from multiple infection types and genera. Complex **2** is a genuine broad-spectrum antimicrobial displaying comparable activity against clinical isolates of resistant *S. aureus* strains in physiologically relevant media. Unlike many previously reported Ru^II^-based complexes, **2** even retains its potent activity against MRSA strains, and in media and pHs that represent a range of physiologically relevant environments, it continues to display efficacy against both Gram positive and Gram negative bacteria. Encouragingly, cotreating bacteria with complex **2** and established antibiotics or its dinuclear analogue, **2** produced no antagonistic effects but consistently resulted in markedly additive effects, which in the case of Meropenem borders into synergy. Furthermore, a single dose of the complex directly images and completely clears an AMR strain of *A. baumannii* from *G. mellonella*, an infection the intrinsic immune system of the model organism is incapable of clearing by itself. This successful treatment has no detectable deleterious effect on *Galleria* larvae and requires concentrations that are considerably lower than those tolerated by this model. Interestingly, total clearance of the bacteria occurs in <48 h, which is considerably faster (∼96 h) than equivalent treatment with **1**. This effect and the observation that it continues to be active during bacterial early exponential growth phase, means that **1** may be suited to shorter treatment courses, a type of regime that has been identified as optimizing cure rates and minimizing the potential for bacterial resistance acquisition.^94,95^

Taken together, the findings in this study provide clear evidence that complex **2** offers considerable potential as a novel broad-spectrum antimicrobial. Although a previous report has established that this complex targets bacterial nucleic acids,^65^ extensive experiments to delineate the exact details of its mechanism of action at a molecular level are currently underway and these will form the basis of a forthcoming study.

## 4 Experimental Section

### 4.1 Bacterial Strains and Growth

Growth was achieved in Mueller–Hinton broth, glucose containing defined minimal medium,^62^ chemically defined medium^64^ plasma-like medium,^72,73^ or artificial urine medium^71^ made up as per manufacturer’s instructions or as described in the literature using standard techniques. Strains were obtained from The McLean culture collection, Chesterfield Royal Hospital, UK and Nottingham University Hospitals Trust Pathogen Bank, UK.

### 4.2 Preparation and Storage of Complex **2**

The complex was synthesized as previously described.^65^ Stock solutions were made to a concentration of 5 mg mL^–1^ in sterile deionized water and stored at room temperature and protected from light.

### 4.3 Crystal Structure of **2**

X-ray quality crystals of [**2**]Cl_2_ were grown by slow evaporation of an ethanol/acetone solution. Details of its empirical formula and structural refinement are in Table 4. More details of bond angles and lengths are available in the CIF file (CCDC 2354069) deposited with the Cambridge Crystallographic Data Centre.

**Table 4:** Crystal Data and Structure Refinement

identification code	iaj713k_0m
empirical formula	C_59_H_56_Cl_2_N_10_O_3_Ru
formula weight	1125.10
temp, K	100
crystal system	triclinic
space group	*P*1̅
*a*, Å	13.6538(16)
*b*, Å	14.1267(17)
*c*, Å	15.5268(18)
α, deg	98.318(3)
β, deg	93.030(3)
γ, deg	92.277(4)
vol, Å^3^	2955.8(6)
*Z*	2
ρ_calc_, g/cm^3^	1.264
μ, mm^–1^	0.406
*F*(000)	1164.0
crystal size, mm^3^	0.4 × 0.35 × 0.15
radiation	Mo Kα (λ = 0.71073)
2θ range for data collection, deg	5.346 to 56.926
index ranges	–18 ≤ *h* ≤ 18, −18 ≤ *k* ≤ 18, −20 ≤ *l* ≤ 20
reflections collected	64,507
independent reflections	14,811 [*R*_int_ = 0.0472, *R*_sigma_ = 0.0449]
data/restraints/params	14,811/661/700
goodness-of-fit on *F*^2^	1.058
final *R* indexes [*I* ≥ 2σ(I)]	*R*_1_ = 0.0567, w*R*_2_ = 0.1557
final *R* indexes [all data]	*R*_1_ = 0.0763, w*R*_2_ = 0.1717
largest diff. peak/hole, e Å^–3^	1.17/–0.88

### 4.4 Bioinformatic Analysis of *S. aureus* Strains

Genomic DNA was extracted from cell pellets using a genomic DNA isolation kit (Merck) as per manufacturer’s instructions. DNA quality was checked using a NanoDrop microvolume spectrophotometer (ThermoFisher Scientific) and quantified using a Qubit 4 fluorometer (Invitrogen) high sensitivity dsDNA assay kit. Strains were sequenced on the Illumina HiSeq/NovaSeq platform and assembled by a MicrobesNG (Birmingham, UK). General features of the isolates are summarized in Table S5. The genomic data generated during this study are available in the National Center for Biotechnology Information BioProject: PRJNA1069650. BioSample accession numbers: SAMN39618626-36. SRR raw Illumina data: SRR28824215-25. Annotated genomes are available in figshare. Antimicrobial resistance markers were identified using Resistance Gene Identifier (RGI) v6.0.0 tool of the Comprehensive Antibiotic Resistance Database (CARD) v3.2.5.^96^ Only resistance genes that showed a perfect or strict match with coverage for a given gene and achieved ≥90% identity and read length in the database were included in this study. The Virulence Factor Database platform VFAnalyzer was used to predict virulence factors present within the draft genomes.^97^ Phage elements were predicted using PHASTEST^98^

### 4.5 Minimal Inhibitory and Bactericidal Assays

MIC assays were performed as previously described. Following the MIC assay, 10 μL from each well displaying no visible growth was transferred to an MHA plate, along with a positive growth control. Plates were incubated for 18 h at 37 °C. The lowest concentration showing no growth was recorded as the MBC.

### 4.6 Growth Inhibition Assays

1 mL of growth medium was added to wells in triplicate per condition. A 1% overnight culture grown in the same medium was added to each well, excluding sterility control wells. A polyurethane Breath-Easy membrane (Merck) was applied and plates were incubated shaking using a double orbital at and 37 °C in a Cytation 3 plate reader set to take OD_600_ reads every 15 min for 24 h. For assays with compound addition, when cultures reached early exponential phase (OD_600_ ∼ 0.4) plates are removed varying concentrations of compound were added, a new Breath-Easy membrane was applied and plates were reincubated with measuring OD_600_ every 15 min for a further 24 h.

### 4.7 Checkerboard Synergy Assays

Checkerboard microdilution assays were set up as previously described. FIC index (FICi) values were determined for each drug combination, with synergy recorded where the FICi < 0.5, additive recorded where the FICi value was 0.5–4, and antagonism recorded for combinations with a FICi value of >4.^99^

### 4.8 *G. mellonella* Assays

TruLarv *G. mellonella* were used for this study to ensure they were reared without antibiotics and were all a similar weight. For each compound concentration, seven larvae were used, and for the control, 15 larvae were used. Insects were injected on the initial day with 10 μL of the correct concentration stock solution of **2** or water (control) into their left pro-leg. Once injected larvae were stored in a Petri dish containing filter paper and incubated at 37.5 °C. Three analysis tests were conducted at 0, 24, 48, 72, 96, and 120 h. Activity scores were recorded: 0-no movement, 1-corrects itself, 2-movement on stimulation, and 3-movement without stimulation. Live/dead scores were recorded to produce percentage survival curves. Melanization was scored on a scale of 0–4: 0—completely black, 1—black spots, 2—tail/line black and 4—none. Cocoon formation was not observed in this case. At the end of the toxicity screen *Galleria* larvae were disposed of in a humane manner.^100^

### 4.9 Transmission Electron Microscopy

Cells were fixed using 3% glutaraldehyde. Cells were dehydrated using a series of ethanol washes (70–100% ethanol) and TEM samples sectioned in Araldite resin by microtome. Samples were examined on an FEI Tecnai instrument operating at 80 kV equipped with a Gatan 1 K CCD camera. Images were processed and analyzed using FIJI ImageJ software.

## Supporting Information Available

Further MIC data for clinical isolates of Gram negative pathogens and more CAU(TI) isolates, prophage predictions for *S. aureus* clinical isolates determined using PHASTEST, MIC, and MBC values for clinical isolates of resistant *S. aureus* clinical isolates, summary information for genomes generated from the *S. aureus* isolates and associated predicted virulence factors according to the Virulence Factor Database, summary of sensitivity of *E. coli* EC958 to clinically employed antibiotics, and Kaplan–Meier percentage survival curves for the AB184 infection model in untreated and treated *Galleria* (PDF)
HPLC traces of complex **2** (PDF)

## Abbreviations

AMR: antimicrobial resistance
AUM: artificial urine medium
(CA)UTI: (Catheter Associated)
CRAB: carbapenem resistant *A. baumannii*
EUCAST: European Committee on Antimicrobial Susceptibility Testing
gDMM: glucose-defined minimal medium
MRSA: Methicillin-resistant *Staphylococcus aureus*
UTI: urinary Tract Infection
PLM: plasma-like medium.
