# Supplementary material for: In Vitro and In Vivo Studies on a Mononuclear Ruthenium Complex Reveals It is a Highly Effective, Fast-Acting, Broad-Spectrum Antimicrobial in Physiologically Relevant Conditions
Source: ACS Infect Dis. 2024 Aug 6;10(9):3346–57. doi: 10.1021/acsinfecdis.4c00447 (PMC11406528; doi:10.1021/acsinfecdis.4c00447)
Supplement: Supplementary file 1 — id4c00447_si_001.pdf [file id4c00447_si_001.pdf]

Supplementary Information

In vitro and *in vivo* studies on a mononuclear ruthenium complex reveals it is a highly effective, fast-acting, broad-spectrum antimicrobial in physiologically relevant conditions **-** *Supplementary material***.**

Adam M. Varney,[ab] Kirsty L. Smitten [c,d] Hannah M. Southam,[d] Simon D. Fairbanks,[c] Craig C. Robertson,[c] Jim A. Thomas[c] and Samantha McLean*[a].*

[a]School of Science and Technology, Nottingham Trent University, Clifton Lane, Nottingham, NG11 8NS, UK.

[b]Medical Technologies Innovation Facility (MTIF), Clifton Lane, Nottingham, NG11 8NS UK.

[c]Department of Chemistry, University of Sheffield, Brook Hill, Sheffield, S3 7HF, UK.

[d]School of Bioscience, The University of Sheffield, Western Bank, Sheffield, S10 2TN, UK

Table S1. RuTMP is effective in inhibiting a range of clinically isolated Gram-negative pathogens

Strain	Origin	MIC (µM)
P. aeruginosa	Neonatal sepsis (SJF 2304)	7.64 ± 1.13
K. pneumoniae	Neonatal enterocolitis (SJF 2312)	6.25 ± 0.00
S. enterica	Human isolate (SJF 1234)	3.47 ± 0.28
E. hormaechei	Cerebrospinal fluid (SJF 1439)	5.56 ± 0.57
C. koseri	Neonatal meningitis (SJF 2294)	4.17 ± 0.49
A. baumannii	Human isolate (SJF 1183)	6.95 ± 0.57
S marcescens	NUH (21Y000040)	3.16 ± 0.00
A. baumannii	Human isolate AB184	2.34 ± 0.78
E. coli 12241	ATCC 25922	3.82 ± 0.28

N=3 ± S.D. Strains were obtained from the Steven Forsythe culture collection, Nottingham Trent University, the ATCC, Nottingham University Hospital (NUH) or were part of the University of Sheffield culture collection. MIC assays were performed in defined minimal medium containing glucose.

Table S2. Standard deviation for Table 1 MIC and MBC assays of the (CA)UTI isolates

SD Table CA(UTI) isolates												
**Medium →**	**gDMM**				**Plasma-Like medium**				**Artificial Urine Medium**			
**Compound →**	**Dinuclear**		**Mononuclear**		**Dinuclear**		**Mononuclear**		**Dinuclear**		**Mononuclear**	
**Strain ↓**	**MIC**	**MBC**	**MIC**	**MBC**	**MIC**	**MBC**	**MIC**	**MBC**	**MIC**	**MBC**	**MIC**	**MBC**
E. coli MG1655	0.37	0.00	0.00	0.00	0.00	2.95	0.37	0.00	0.00	0.00	0.00	5.89
***E. coli* EC958**	0.00	0.00	0.00	1.47	0.00	1.47	0.00	0.00	0.00	5.89	0.00	11.79
***K. pneumoniae*[18Y000138]**	0.37	2.42	1.47	0.00	1.47	5.89	0.00	0.00	0.00	5.89	0.00	0.00
***K. pneumoniae*[18Y001710]**	0.37	1.95	0.00	1.47	0.00	2.95	0.00	5.89	0.00	0.00	0.00	11.79
***E. asburiae*[18Y001733]**	0.00	0.00	0.00	1.47	0.00	7.80	0.00	17.68	0.00	5.89	0.00	11.79
***E. cloacae*[19Y000094]**	0.00	3.90	0.00	2.95	0.00	0.00	0.00	0.00	0.00	5.89	0.00	11.79
***E. cloacae*[19Y000373]**	0.37	4.83	0.74	1.47	0.74	0.00	1.47	0.00	0.00	0.00	0.00	17.68
***E. coli*[20Y000092]**	N/A	N/A	N/A	N/A	0.00	2.95	0.00	0.00	0.74	0.00	0.00	11.79

All data presented in µM.

Table S3. Prophage predictions of ten *S. aureus* clinical isolates, determined using PHASTEST

Strain	Completeness	Region Length	GC %	# Total Proteins	Most Common Phage
S. aureus USA300	incomplete	20.6Kb	30.80%	20	PHAGE_Staphy_PT1028_NC_007045(5)
	incomplete	7Kb	26.08%	17	PHAGE_Strept_9873_NC_047763(1)
	intact	60.4Kb	32.69%	73	PHAGE_Staphy_phi2958PVL_NC_011344(29)
	intact	75.3Kb	31.73%	76	PHAGE_Staphy_P282_NC_048634(27)
S. aureus W116727	incomplete	7.7Kb	26.49%	18	PHAGE_Staphy_StB27_NC_019914(2)
	incomplete	31Kb	29.25%	27	PHAGE_Staphy_StauST398_5_NC_023500(2)
	intact	46.4Kb	32.98%	66	PHAGE_Staphy_SA1014ruMSSAST7_NC_048710(22)
S. aureus W116752	intact	45.1Kb	33.03%	66	PHAGE_Staphy_StauST398_4_NC_023499(38)
S. aureus W116753	intact	49Kb	34.77%	69	PHAGE_Staphy_3MRA_NC_028917(45)
	intact	49Kb	34.77%	69	PHAGE_Staphy_3MRA_NC_028917(45)
S. aureus W116759	incomplete	19.8Kb	29.91%	29	PHAGE_Staphy_187_NC_007047(4)
	intact	45.9Kb	32.85%	65	PHAGE_Staphy_SA345ruMSSAST8_NC_048713(25)
S. aureus W116765	incomplete	19.8Kb	29.91%	29	PHAGE_Staphy_187_NC_007047(4)
	intact	45.9Kb	32.85%	65	PHAGE_Staphy_SA345ruMSSAST8_NC_048713(25)
MRSA W116067	incomplete	20.6Kb	30.80%	20	PHAGE_Staphy_PT1028_NC_007045(5)
	incomplete	7Kb	26.09%	17	PHAGE_Strept_9871_NC_031069(1)
	intact	60.4Kb	32.69%	73	PHAGE_Staphy_phi2958PVL_NC_011344(29)
MRSA W116587	intact	57.4Kb	32.00%	68	PHAGE_Staphy_phiN315_NC_004740(43)
MRSA M520633	intact	64.8Kb	33.88%	64	PHAGE_Staphy_phiJB_NC_028669(28)
	intact	64.3Kb	32.06%	74	PHAGE_Staphy_SA345ruMSSAST8_NC_048713(33)
MRSA M521259	incomplete	7Kb	26.08%	17	PHAGE_Staphy_55_NC_007060(1)
	questionable	32.7Kb	32.79%	17	PHAGE_Staphy_SPbeta_like_NC_029119(5)
	intact	70.7Kb	31.67%	79	PHAGE_Staphy_P282_NC_048634(58)
MRSA M521385	incomplete	30.1Kb	32.25%	32	PHAGE_Staphy_3A_NC_007053(11)
	intact	32.8Kb	33.43%	37	PHAGE_Staphy_phi2958PVL_NC_011344(21)
	intact	60.5Kb	32.26%	77	PHAGE_Staphy_IME1361_01_NC_048657(36)
	intact	25.9Kb	36.44%	29	PHAGE_Staphy_phiETA_NC_003288(11)
	incomplete	22.7Kb	33.17%	30	PHAGE_Staphy_phiNM_NC_008583(7)

Intact (score > 90) Questionable (score 70-90) Incomplete (score < 70). See PHASTEST website for score classification criteria.[1]

Table S4. Standard deviation for Table 2 MIC and MBC assays of the *S. aureus* isolates

	**SD Table *S. aureus* isolates**			
**Medium →**	**Chemically Defined Medium**		**Plasma-Like Medium**	
**Strain ↓**	**MIC (μM)**	**MBC (μM)**	**MIC (μM)**	**MBC (μM)**
EUCAST *S. aureus* ref	0	2.34375	0	0.74
S. aureus USA300	0	1.5625	0	0.00
S. aureus W116727 (45)	0	1.5625	0	0.00
S. aureus W116752 (46)	0.184142	0.911458	0.048828	9.20
S. aureus W116753 (47)	0.184142	1.5625	0.03069	0.00
S. aureus W116759 (48)	0	1.5625	0.048828	1.33
S. aureus W116765 (49)	0	6.25	0.092071	1.47
MRSA W116067 (50)	0	1.5625	0	2.24
MRSA W116587 (51)	0	1.5625	0.092071	2.66
MRSA M520633 (52)	0.368285	1.5625	0.159472	2.24
MRSA M521259 (53)	0	1.5625	0	1.47
MRSA M521385 (54)	0	0.911458	0	0.74

Table S5. Summary information for genomes generated from the *S. aureus* isolates described in this study

Strain origin /designation	Species identification	Isolated	Accession	Coverage (x)	Length (bp)	Contigs	GC (%)	N50	CDS
USA300 (blood stream)	*S. aureus*	*N/A*	*SAMN39618626*	99x	2845738	19	32.65	872985	2642
W116727 (wound isolate)	*S. aureus*	*14/06/2021*	*SAMN39618627*	78x	2740349	20	32.69	319500	2506
W116752 (wound isolate)	*S. aureus*	*14/06/2021*	*SAMN39618628*	70x	2671053	19	32.83	386063	2452
W116753 (wound isolate)	*S. aureus*	*14/06/2021*	*SAMN39618629*	85x	2751014	25	32.77	378525	2543
W116759 (wound isolate)	*S. aureus*	*14/06/2021*	*SAMN39618630*	109x	2686126	24	32.67	571350	2450
W116765 (wound isolate)	*S. aureus*	*14/06/2021*	*SAMN39618631*	187x	2699385	14	32.77	596684	2507
W116067 (wound isolate)	MRSA	*14/06/2021*	*SAMN39618632*	115x	2879829	26	32.62	525677	2688
W116587 (wound isolate)	*S. aureus*	*14/06/2021*	*SAMN39618633*	81x	2702000	17	32.73	696897	2478
M520633 (Nose and Groin swab)	MRSA	*14/06/2021*	*SAMN39618634*	102x	2804166	15	32.7	468343	2600
M521259 (Nose and Groin swab)	MRSA	*14/06/2021*	*SAMN39618635*	69x	2756252	25	32.65	324789	2538
M521385 (Nose and Groin swab)	MRSA	*14/06/2021*	*SAMN39618636*	109x	2874082	51	32.71	153747	2701

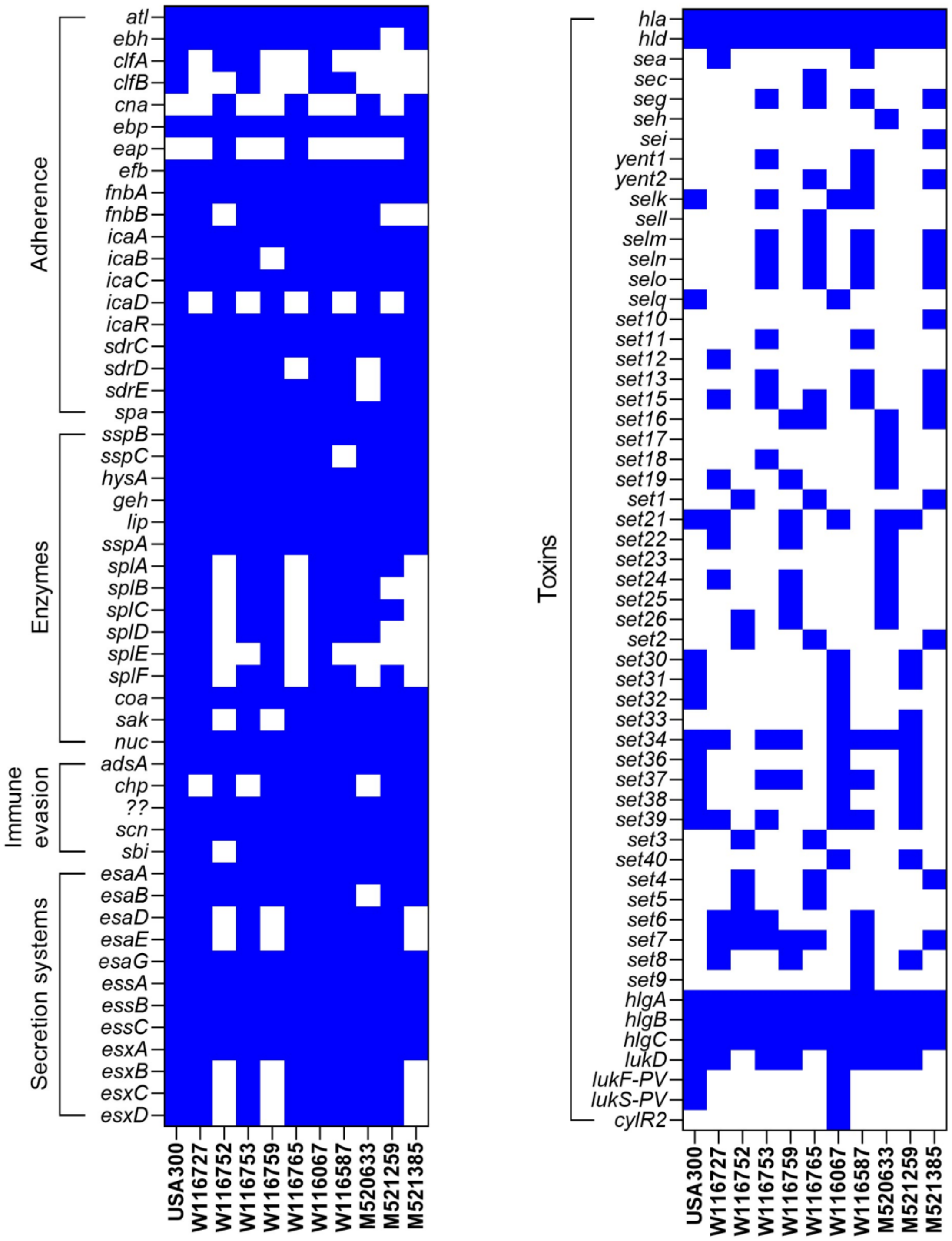

Figure S1: Predicted virulence factors identified in clinically isolated *S. aureus* by whole genome sequencing according to the Virulence Factor Database[3]

Table S6. Sensitivity of *E. coli* EC958 to five common clinically employed antibiotics.

Growth Medium →	gDMM		MHB		
Antibiotic ↓	MIC (mg L^{-1})	MBC (mg L^{-1})	MIC (mg L^{-1})	MBC (mg L^{-1})	Resistance
Cephalexin	128 ± 0	383 ± 34	512 ± 0	2048	✔
Ampicillin	2048 ± 0	4096 ± 0	4096 ± 0	<4096	✔
Ciprofloxacin	256 ± 0	4096 ± 0	4096 ± 0	<4096	✔
Meropenem	0.046 ± 0.01	0.688 ± 0	0.031 ± 0.01	0.059 ± 0.01	✖
Nitrofurantoin	0.75 ± 0.20	3.00 ± 0	2.00 ± 0.00	2.00 ± 0	✖

Antibiotic sensitivity determined via EUCAST MIC assay according to guidelines.[2]

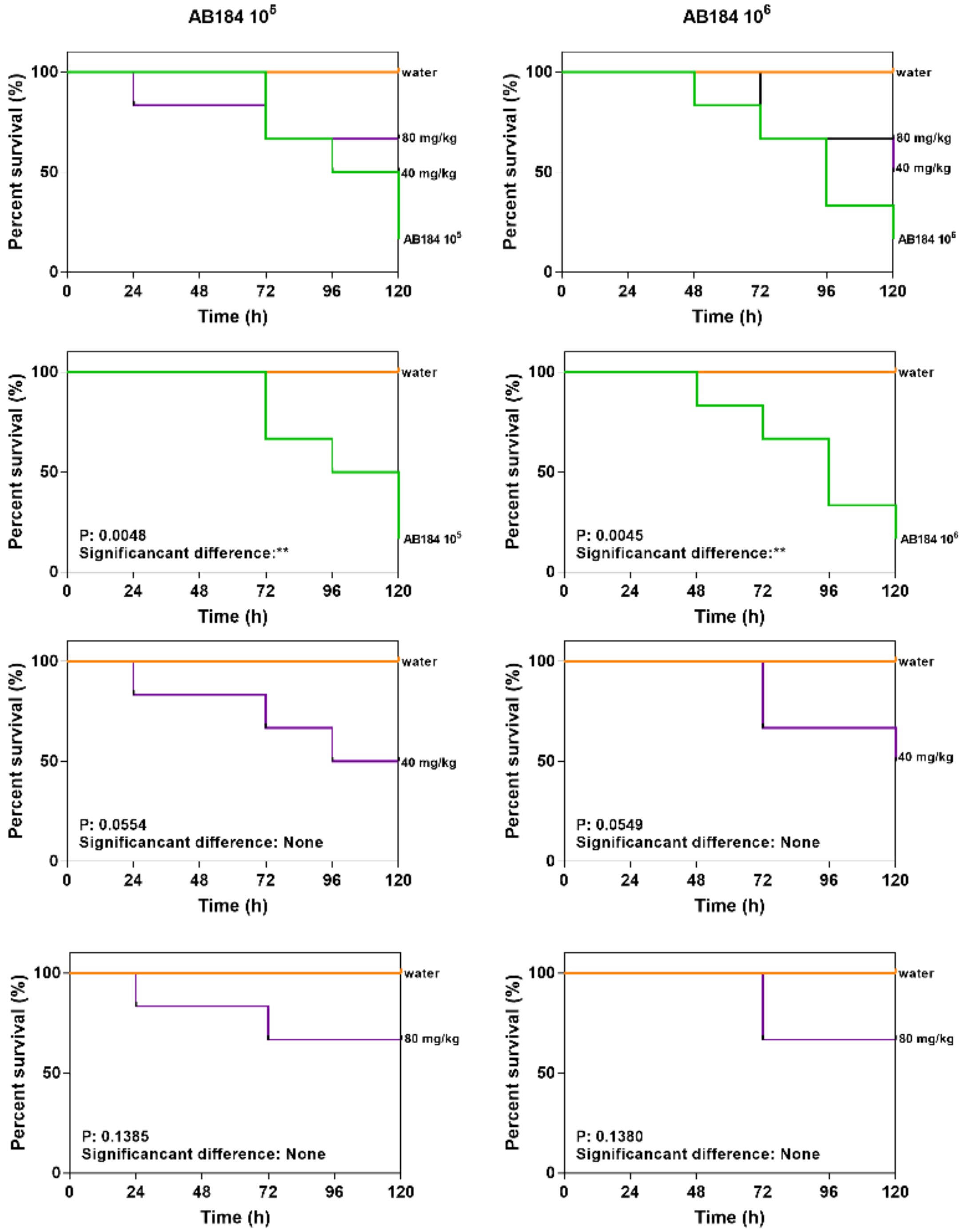

Figure S2. *Galleria Mellonella* infection model. Kaplan Meier percentage survival curves comparing Galleria injected with AB184 (green), water (orange) and **2** (purple) [top]. Percentage survival for AB184 (10^5, 10^6) compared with water [second], 40 mg/kg treatment and water [third] and 80 mg/kg and water [bottom]. Co-injected larvae were injected with bacteria into their right pro-leg then 30 minutes later into their left pro-leg. Larvae were incubated for 120 hours at 37.5*°C*.

References

(1) Wishart, D. S.; Han, S.; Saha, S.; Oler, E.; Peters, H.; Grant, J. R.; Stothard, P.; Gautam, V. PHASTEST: Faster than PHASTER, Better than PHAST. *Nucleic Acids Res.* **2023**, *51* (W1), W443–W450.

(2) https://www.eucast.org/clinical_breakpoints Accessed: 9th May 2024.

(3) Liu B, Zheng D, Zhou S, Chen L, Yang J. VFDB 2022: a general classification scheme for bacterial virulence factors. *Nucleic Acids Res*. 2022, *50(D1)*, D912-D917.
