# Supplementary material for: In Vitro and In Vivo Studies on a Mononuclear Ruthenium Complex Reveals It is a Highly Effective, Fast-Acting, Broad-Spectrum Antimicrobial in Physiologically Relevant Conditions
Source: ACS Infect Dis. 2024 Aug 6;10(9):3346–57. doi: 10.1021/acsinfecdis.4c00447 (PMC11406528; doi:10.1021/acsinfecdis.4c00447)
Supplement: Supplementary file 2 — id4c00447_si_002.pdf [file id4c00447_si_002.pdf]

==== Shimadzu LCsolution Analysis Report ====

<Chromatogram>

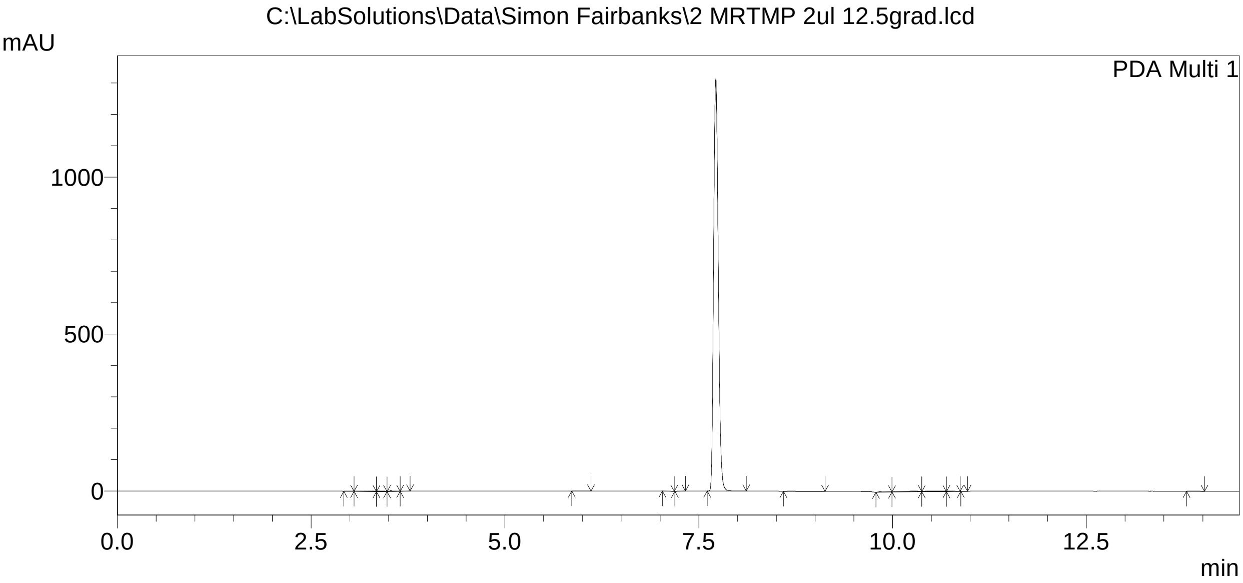

1 PDA Multi 1/254nm 4nm

<Results>

PeakTable

PDA Ch1 254nm 4nm

Peak#	Ret. Time	Area	Height	Area %	Height %
1	2.940	7435	1086	0.131	0.082
2	3.275	23103	1584	0.407	0.120
3	3.400	12722	1855	0.224	0.140
4	3.528	16803	2469	0.296	0.187
5	3.704	3767	574	0.066	0.043
6	5.946	1082	211	0.019	0.016
7	7.077	3273	406	0.058	0.031
8	7.242	3655	759	0.064	0.057
9	7.719	5468168	1302224	96.329	98.610
10	8.666	10876	766	0.192	0.058
11	9.960	25994	2675	0.458	0.203
12	10.274	54448	2255	0.959	0.171
13	10.553	31816	1675	0.560	0.127
14	10.758	9576	1195	0.169	0.090
15	10.932	1184	352	0.021	0.027
16	13.878	2655	493	0.047	0.037
Total		5676556	1320581	100.000	100.000
